# Twenty-seven year surveillance of blood transfusion recipients infected with HIV-1 in Hebei Province, China

**DOI:** 10.1371/journal.pone.0202265

**Published:** 2018-08-15

**Authors:** Suliang Chen, Xinli Lu, Guangyi Bai, Yuqi Zhang, Baojun Li, Wei Wang, Liang Liang, Lin Ma, Yan Li, Xiaofeng Wang, Yingying Wang, Cuiying Zhao, Hongru Zhao

**Affiliations:** Hebei Provincial Center for Disease Control and Prevention, Shijiazhuang, Hebei, China; University of Cincinnati College of Medicine, UNITED STATES

## Abstract

We conducted an investigation of blood management in which blood transfusion recipients underwent molecular biological analysis, to trace the possible source of HIV infection. Epidemiological investigation was carried out among HIV-infected individuals. Blood transfusion recipients infected with HIV were tracked for the date of transfusion, reason for transfusion, hospital where transfusion was received, source of blood, components of transfusion, number of transfusions, and transfusion volume. A total of 285 blood transfusion recipients infected with HIV-1 were detected in Hebei over the study period, with 42.81% (122/285) detected through clinical diagnostic testing. These cases showed a concentrated distribution in southern Hebei, with local outbreak characteristics. A census of the population in Shahe County, which had a high concentration of cases, revealed that recipients of blood transfusions had an HIV infection rate of 15.54% (92/592). Post-transfusion infection frequently occurred among blood transfusion recipients at township medical institutions, with a peak in 1995. Owing to late detection of HIV infection among blood transfusion recipients, the rates of spousal transmission and mother-to-child transmission reached 20.87% and 28.05%, respectively. Around 1995, community medical institutions did not screen for HIV antibodies among paid blood donors, which was an important cause of the outbreak of HIV-1 infection among blood transfusion recipients. Our findings indicate that cases of blood transfusion-related infection decreased rapidly with gradual improvement in the HIV screening system for blood donors that began in 1995, particularly after full implementation of HIV nucleic acid testing of volunteer blood donors was begun in 2015.

## Introduction

Hebei is a province in northern China that surrounds Beijing and Tianjin. It borders Liaoning, Inner Mongolia, Shanxi, Henan, and Shandong provinces and has a population of 74.24 million. The first patient with acquired immune deficiency syndrome (AIDS) in Hebei was found in 1989 [1], and a total of 8233 cases of human immunodeficiency virus (HIV)/AIDS had been reported in the province as of 2015. Around the year 1995, a local epidemic of HIV infection occurred among paid blood donors on the Central Plain of China [2]. Before the Blood Donation Law of China was put into effect in 1998, blood management was disordered and community medical institutions did not properly implement HIV screening systems among blood donors. Therefore, this situation caused a risk of HIV infection for recipients of blood transfusions [3]. Over the years, Hebei Province has been carrying out surveillance and research on HIV infection among blood transfusion recipients, and a considerable amount of epidemiological testing data have been accumulated. We analyzed data of blood transfusion recipients infected with HIV based on transfusion history and an investigation of blood management in Hebei, to trace possible sources of infection. The results of our analysis are reported below.

## Materials and methods

### Ethics statement

Written informed consent was obtained from all participants enrolled in this study. This study was approved by the local Ethics Committee at Hebei Provincial Center for Disease Control and Prevention (CDC).

### Case definition and surveillance

HIV antibody-positive individuals were defined as blood transfusion cases infected with HIV if they met the following criteria: 1) history of having received blood and blood products; 2) no factors related to sexual activity, intravenous drug use, and intrafamilial transmission; and 3) current residence within the territory of Hebei Province. The basic information of all cases was derived from the database of the National Information System for Comprehensive HIV/AIDS Prevention and Control in China. Hebei Province has 600 HIV testing laboratories, 2271 rapid testing points, 521 voluntary counseling and testing points, and 12 western blot laboratories at various levels of medical and health care institutions and blood centers in the province. An expanded free testing policy has been advocated in Hebei, and epidemiological investigation has been carried out among HIV-infected individuals. Collected information is entered into the national database and HIV-infected cases are regularly followed up. Blood transfusion recipients infected with HIV are tracked for the date of transfusion, reason for transfusion, hospital where transfusion was received, source of blood, components of transfusion, number of transfusions, and transfusion volume.

### Outbreak investigation

A number of blood transfusion recipients were infected with HIV in Shahe County over the period 1999–2002. To determine the HIV infection rate among local recipients of blood transfusions, a local population census was conducted from November 2003 to February 2005. All individuals who had a history of transfusion between January 1, 1994 and December 31, 1998 were included in the epidemiological case investigation, and blood specimens were collected for HIV antibody screening.

### Investigation of intrafamilial transmission

For blood transfusion recipients infected with HIV who had a spouse, those who were pregnant, had given birth, or who were breastfeeding after blood transfusion, infection factors other than spousal transmission and mother-to-child transmission (MTCT) were excluded for the spouses and children. Spouses and children were included in the investigation of spousal transmission and MTCT, and their blood specimens were obtained for HIV antibody testing.

### Natural history of cases

HIV-infected individuals and patients who received only one blood transfusion without antiviral therapy were observed during the latency and survival periods. The latency period was considered to be the time between the date of transfusion and date of onset; the survival period was the time between the date of onset and date of death owing to AIDS. The earliest detection date of CD4 <200 cells/mm^3^ was defined as the date of onset according to the Diagnostic Criteria for HIV/AIDS (WS 293–2008) [4]. The incidence rate was calculated as the number of patients divided by the number of person-years of observation; the mortality rate was calculated as the number of deaths owing to HIV/AIDS divided by the number of person-years of disease during the observation period. Observation ended on December 31, 2010.

### HIV antibody testing and subtyping

Primary screening was conducted using ELISA reagents for the detection of antibodies to HIV type 1 (HIV-1) and HIV type 2 (HIV-2). Blood specimens from HIV-positive individuals were subjected to a confirmatory test by western blot assay (HIV Blot 2.2; MP Biomedicals Asia Pacific Pte. Ltd, Singapore), and positive specimens were considered to be HIV-positive cases. In this study, a total of 37 HIV-1 *pol* gene sequences were obtained from 285 blood specimens with paid blood and post-transfusion HIV infection. These blood specimens with *pol* sequences were collected between 1995 and 2013, and included paid blood donors from Langfang and blood recipients from Xingtai. No spouse- or vertical-paired specimens were obtained owing to death of the mother (or her child) or spouse. We attempted to amplify and sequence HIV-1 *pol* (HXB2: 2147–3462) gene sequences in the reverse transcriptase gene coding region from as many samples as possible. However, sequences were not obtained from all the samples owing to limited blood plasma volume, poor quality of the samples, and limited long-term storage conditions. Maximum likelihood (ML) tree analysis was performed using MEGA 6.0 with 1,000 replicates [5], and HIV genotyping was performed.

### Data analysis

The data under investigation were entered into a database and frequency analysis was conducted using SPSS Version 11.0 (SPSS Inc., Chicago, IL, USA). Survival was analyzed using the life-table method. Both latency and survival periods were estimated using the median method. Medians and 95% confidence intervals (CIs) were calculated using the bootstrap method.

## Results

### General situation of blood transfusion cases

**Fig 1:**
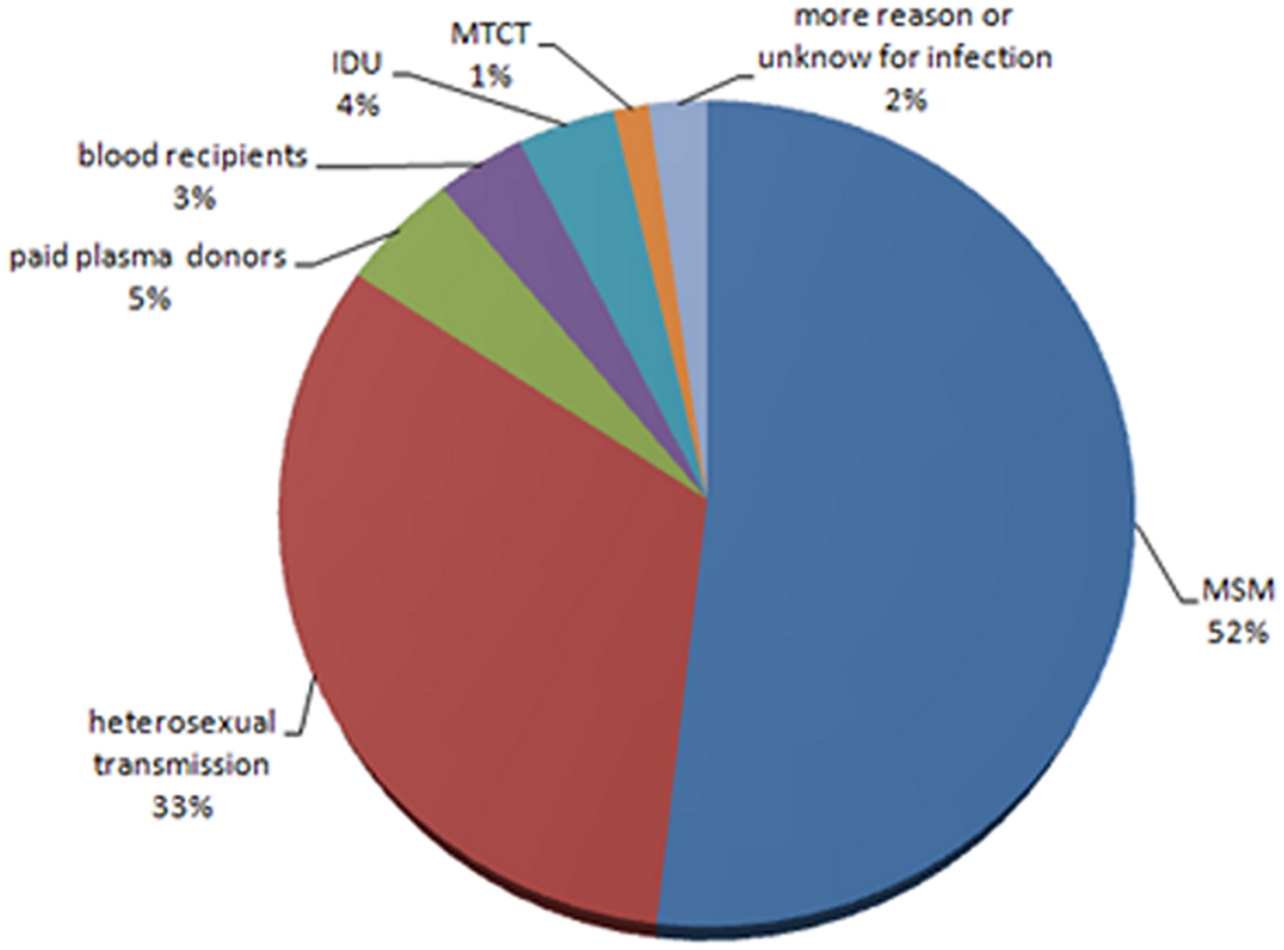
Distribution of transmission routes in Hebei.

In Hebei Province, the first patient with HIV/AIDS was detected on October 27, 1989. The patient was an exported laborer who had just returned from the Democratic Republic of the Congo in Africa. As of 2015, a total of 8233 cases of HIV/AIDS had been reported in Hebei. There were 4267 cases (52.83%) of HIV infection among men who have sex with men, 2678 cases (32.53%) of heterosexual transmission, 389 cases (4.72%) through former paid plasma donation, 285 cases (3.46%) through blood transfusion, 314 cases (3.81%) through injection drug use, 112 cases (1.36%) of MTCT, and 188 cases (2.28%) of multiroute transmission or unknown transmission route. The composition of different transmission routes is illustrated in Fig 1. Testing results showed that all blood transfusion cases and those with transmission of HIV between spouses or from mother to child were infected with HIV-1.

The 285 cases of blood transfusion recipients infected with HIV-1 reported in this study were distributed among 175 administrative villages, 101 towns, 55 counties, and 11 cities of Hebei Province. Cases at the city and county level were mainly distributed in the southern Hebei region. There were 193 women (sex ratio, 2.10:1); 226 farmers; 36 workers, businessmen, service providers, teachers, and retirees; and 9 children and students. A total 284 individuals were Han Chinese and 1 was Mongolian. The youngest age at infection through blood transfusion was 42 days after birth, and the oldest was 61 years old; the mean age at infection through blood transfusion was 27 years.

### Detection methods of blood transfusion cases

Among the 285 cases of blood transfusion recipients with HIV-1, 42.81% (122/285) were detected by clinical diagnostic testing (HIV antibody testing was carried out in the hospital), 17.89% (51/285) by on-site clinical diagnostic testing (HIV antibody testing was carried out at the same time as on-site investigation), 6.84% (48/285) during voluntary counseling and testing, 7.02% (20/285) by preoperative testing, and 3.16% (9/285) by testing at the time of voluntary blood donation.

### Date of transfusion and detection date of HIV infection

**Fig 2:**
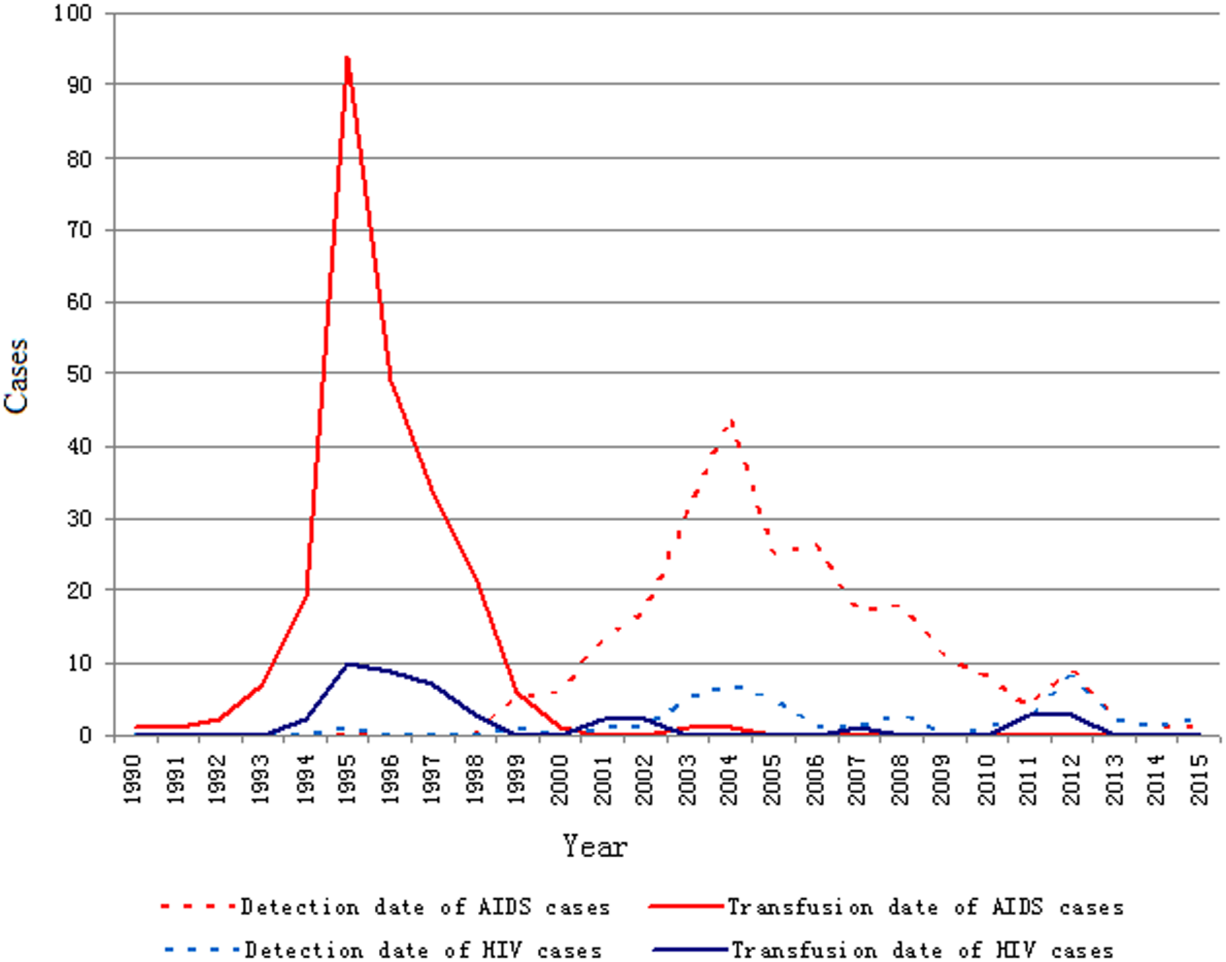
Date of transfusion and detection date of HIV infection.

Five of the 285 cases of blood transfusion recipients infected with HIV-1 had an unknown date of transfusion. For the remaining 280 cases, the earliest date of transfusion was June 1, 1990, and the patient was transfused with whole blood from the blood center in Xingtai city for splenic rupture. The latest date of transfusion was August 31, 2012, and the patient was transfused with whole blood and plasma for burn skin grafting. The peak number of transfusion HIV/AIDS cases was recorded in 1995. The earliest detection date of HIV infection was March 6, 1995 and the latest was November 10, 2015. The peak number of cases detected was recorded in 2004. The distribution of dates of transfusion and detection is shown in Fig 2.

The 280 cases of blood transfusion recipients infected with HIV-1 were reviewed, to obtain the date of transfusion and first detection date of HIV antibodies. The time from transfusion to detection of HIV infection varied from a minimum of 3 months to a maximum of 240 months, with a mean period of 109.5 months. The peak detection time was 60–159 months after transfusion (70.71%), and cases were detected relatively late (Fig 2).

### Location of transfusion

#### Level of hospital at which transfusion took place

The 285 blood transfusion cases involved 65 hospitals, including 48 hospitals in Hebei and 17 hospitals in other provinces. In the territory of Hebei, 81.82% of transfusion cases occurred in township hospitals whereas 17.42% of cases occurred in city and county hospitals. A total of 21 cases occurred at 17 hospitals in other provinces, including 14 cases of transfusion at hospitals in Henan, 2 cases at hospitals in Beijing, 2 cases at hospitals in Heilongjiang, and 1 case each in Shanxi, Sichuan, and Guangdong provinces. Furthermore, 47.62% of blood transfusion cases occurred in city hospitals of other provinces. The level of medical institution at which blood transfusion took place is shown in Table 1.

**Table 1:** Distribution of blood transfusion recipients infected with HIV-1 according to different level hospitals.

Level of hospital	Hospitals from Hebei				Hospitals from other provinces			
	Hospitals	%	Infected cases	%	Hospitals	%	Infected cases	%
Municipal level	12	25.00	15	5.68	6	35.29	10	47.62
County level	18	37.50	31	11.74	5	29.41	5	23.81
Township level	16	33.33	216	81.82	3	17.65	3	14.29
Unknown	2	4.17	2	0.76	3	17.65	3	14.29
Total	48	100.00	264	100.00	17	100.00	21	100.00

#### Location distribution of hospitals at which transfusion took place

After excluding the 21 cases of transfusion that occurred at hospitals in other provinces and 2 cases of transfusion at unknown locations in Hebei Province, we analyzed the geographical distribution of the remaining 262 recipients of blood transfusion within the territory of Hebei. The transfusion location was characterized by a concentrated distribution in southern Hebei.

### Intrafamilial transmission

Among the 285 blood transfusion cases, 264 were married and 81 were associated with spousal transmission or MTCT, with an intrafamilial transmission rate of 30.68% (81/264).

Among the 265 spouses of 264 blood transfusion cases that were married, 254 were tested for HIV antibodies and 53 of them were found to be HIV positive, with an infection rate of 20.87%. There were 182 male spouses, 34 of whom were HIV positive; the chance of transmitting post-transfusion HIV infection from wives to husbands was 18.68%. Meanwhile, there were 72 female spouses, 19 of whom were HIV positive; the chance of transmission from husbands to wives was 26.39%. Before the screening test of HIV antibody, the unprotected sexual activities of blood transfusion recipients infected with HIV resulted in their spouses being infected with HIV. There was no significant difference between the rate of sexual transmission from males to females and females to males (χ2 = 1.86, p>0.10).

Among the 285 blood transfusion cases, 193 were female, including 188 married women. Among these, 125 mothers and 168 children had a risk of MTCT. Four children who refused to provide blood specimens were excluded, and 46 of the remaining 164 children tested positive for HIV antibody, with an MTCT rate of 28.05%. A total 95 patients had a risk of transmission to sons; of these, 28 were HIV positive, with a 29.47% chance of mother-to-son transmission. A total 69 patients had a risk of transmission to daughters; of these, 18 were HIV-positive, with a 26.09% chance of mother-to-daughter transmission. The rate of mother-to-son transmission was not significantly different from that of mother-to-daughter transmission (χ2 = 0.23, p>0.50).

### Natural history of cases

**Fig 3:**
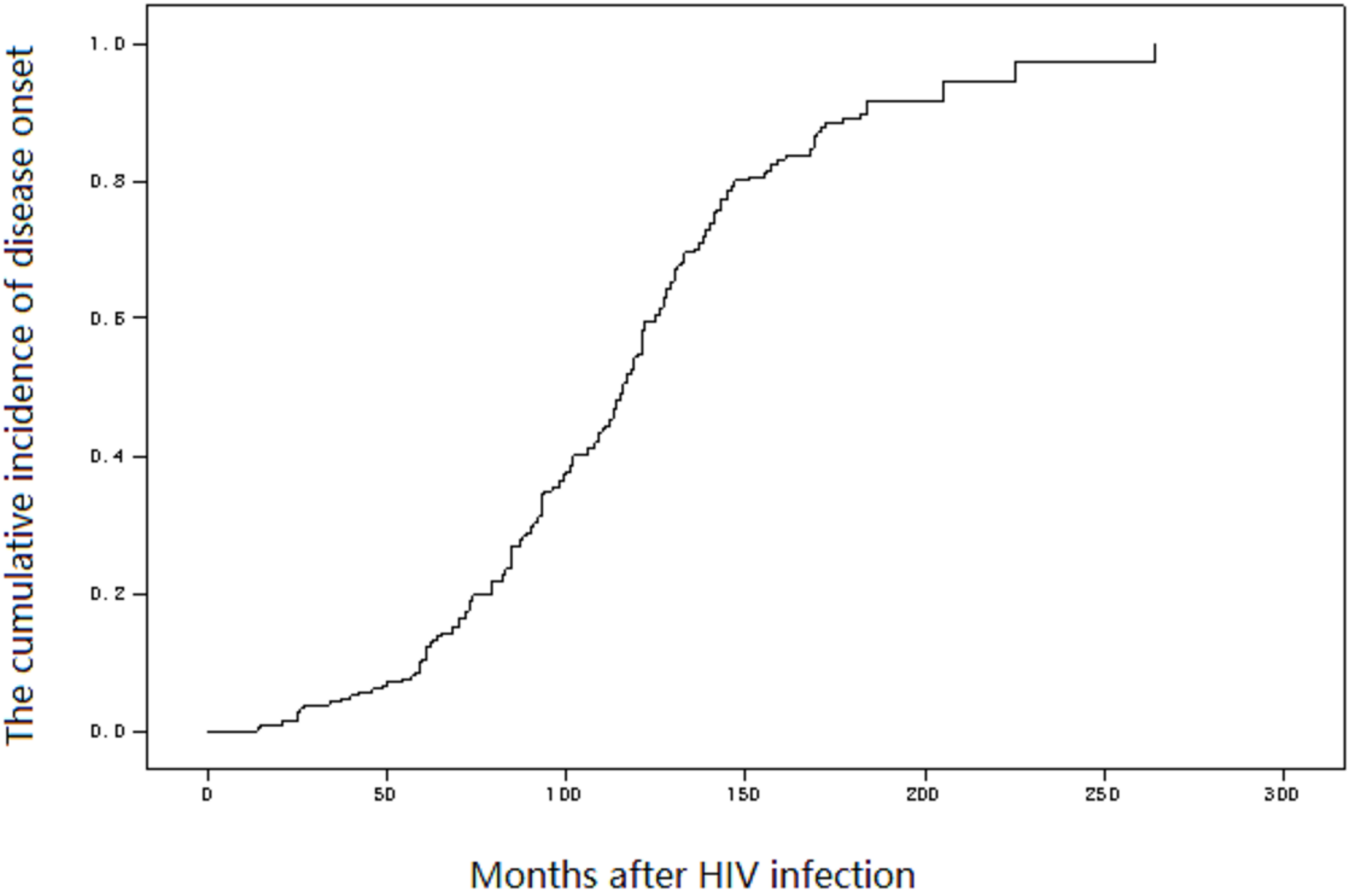
Natural history in blood transfusion recipients infected with HIV-1.

**Fig 4:**
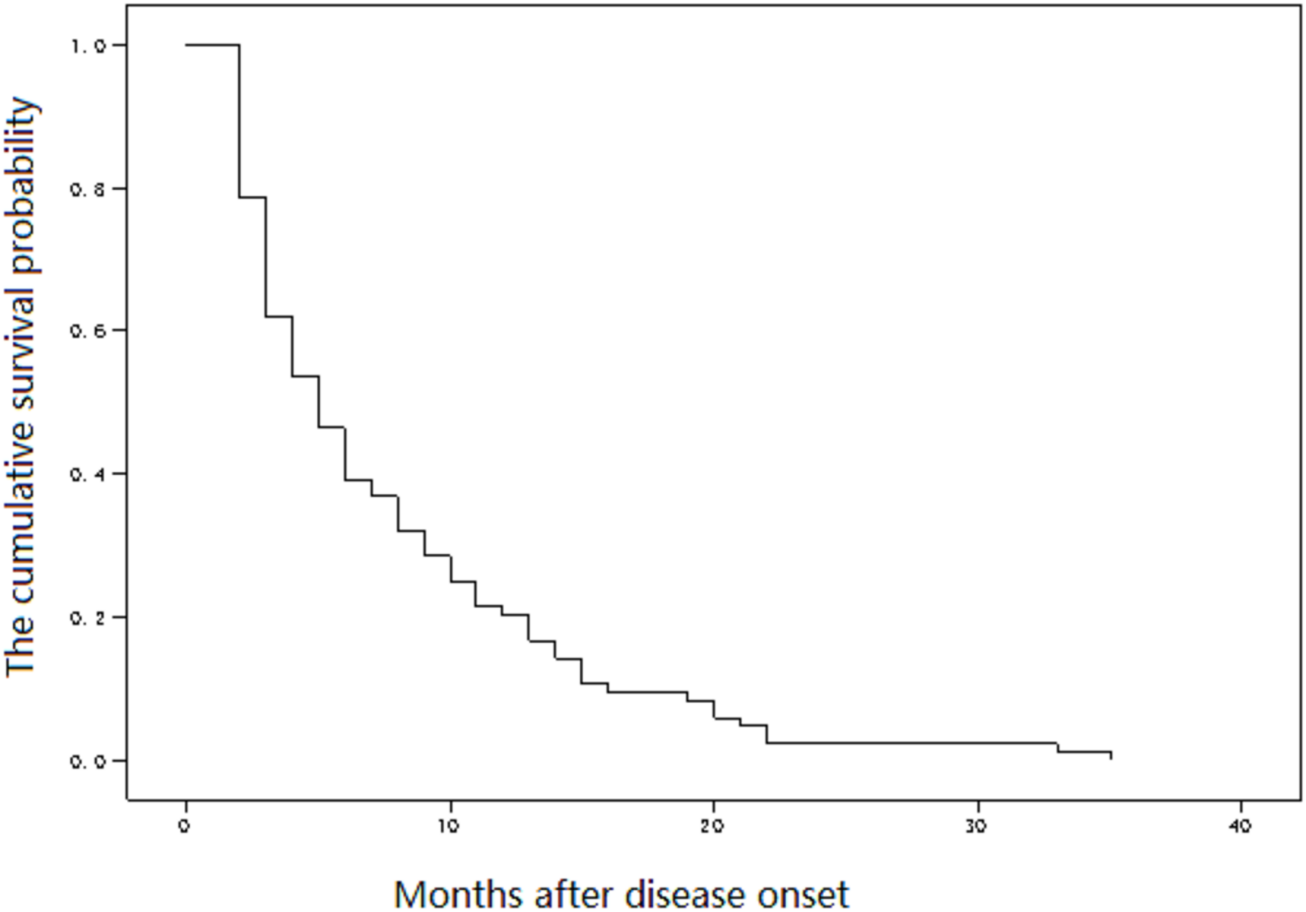
Survival probability in blood transfusion recipients infected with HIV-1.

A total 212 participants under observation during the latency period did not undergo antiviral therapy before disease onset. By the end of observation, the incidence rate was 9.58/100 person-years (191/1993.61). A total 191 cases had onset within 13–263 months after transfusion, with a mean latency time of 115 months (95% CI: 110.11–119.89) (Fig 3). Among the 191 cases with onset of disease, 105 received antiviral therapy; the remaining 86 did not receive any antiviral therapy and all 86 had died by the end of the observation period. According to the Diagnostic Criteria for HIV/AIDS (WS 293–2008) [4], an HIV-positive patient with clinical manifestation or/and CD4 <200 cells/mm^3^ is defined as having HIV/AIDS. Except for 2 cases of non-AIDS deaths, 84 patients with HIV/AIDS died 1–34 months after onset, with a mortality rate of 182.05/100 person-years (84/46.14) and a mean survival of 4 months (95% CI: 2.59–5.49) (Fig 4). Among 105 patients who received antiviral therapy, 19 died within 57 (12–120) months after disease onset, with a mortality rate of 4.42/100 person-years (19/430.16).

### Investigation of the reason for transfusion

Among the 285 cases of blood transfusion recipients infected with HIV, 17 patients received a blood transfusion for blood system diseases (5.96%, including 3 patients with hemophilia, 2 with leukemia, 10 with anemia, and 2 patients with aplastic anemia and thrombocytopenia); 127 patients received transfusion during pregnancy and childbirth (44.56%, including 79 cases of vaginal delivery, 10 cases of ectopic pregnancy surgery, 32 cases of cesarean section, and 6 cases of artificial abortion); 30 patients received transfusion for gynecological diseases (10.53%, including 18 cases of uterine fibroid surgery, 6 cases of hysterectomy, 2 cases of ovarian cancer surgery, 1 case of breast cancer surgery, and 3 cases of other diseases); and 16 patients received transfusion for digestive system diseases (5.61%, including 4 cases of gastrointestinal tumor surgery, 8 cases of peptic ulcer bleeding, and 4 cases of other diseases). A total 79 patients received blood transfusion for trauma (27.72%, including 72 cases of trauma surgery, 3 cases of burns, and 4 cases of amputation surgery); 4 patients received blood transfusion for diseases of the respiratory, endocrine, and circulatory systems and metabolic diseases (1.75%, including 1 case of pneumonia, 2 cases of thyroid surgery, and 1 patient with congenital heart disease); and 12 patients received transfusion for other reasons (4.21%, including 2 cases of poisoning, 1 case of hypoglycemia, and 9 cases for unknown reasons).

### Blood source and transfusion components

We investigated the source of transfused blood among the 285 blood transfusion cases. There were 249 patients (87.37%) transfused with blood that was collected and supplied by medical institutions, 20 (7.02%) with blood from blood centers, 4 (1.40%) with blood purchased from other medical institutions, and 12 patients (4.21%) transfused with blood from unknown sources. Furthermore, we analyzed the components or types of transfused blood. There were 264 patients (92.63%) transfused with whole blood, 6 (2.11%) with plasma, 2 (0.70%) with factor VIII, 2 (0.70%) with whole blood and plasma, and 1 patient (0.35%) transfused with whole blood and platelets. The transfusion volume varied among patients, ranging from 200–25400 mL, with a median volume of 400 mL.

### Outbreak investigation

Based on analysis of the current residence of blood transfusion recipients and their location of transfusion, we found that the largest number of cases occurred in Shahe County of Xingtai City. Cases continuously emerged during 1999–2002. A census of transfusion history was carried out among the entire population of Shahe from November 2003 to February 2005. There were 471,433 people in 15 townships and districts of the county, 452,459 of whom participated in the investigation, with a participation rate of 95.98%. There were 799 recipients of blood transfusion from January 1, 1994 to December 31, 1998, and blood specimens were obtained from 592 of these recipients, with a specimen collection rate of 74.09%. No blood specimens were collected from the remaining 207 blood recipients, who either refused or had relocated from the area. Specimens from the 592 blood recipients were tested for HIV antibodies and 92 were found to be positive, with an infection rate of 15.54%. All infected individuals were local farmers, who denied multiple sexual partners and injection drug use; among the various risk factors for infection, blood transfusion was the likely source of infection for these cases. The distribution of cases at medical institutions where transfusions were received is shown in Table 2. An analysis of the dates of transfusion is presented in Table 3.

**Table 2:** HIV infections among blood recipients from hospitals between 1994 and 1998 in Shahe County.

Groups of hospitals	Hospitals	Recipients	HIV screening	Detection rate (%)	Infected cases	Infection rate (%)
Five hospitals with the history of blood transfusion in Shahe	BT Township hospital	7	6	85.71	3	50.00
	KT Township hospital	95	89	93.68	42	47.19
	XDW hospital	214	174	81.31	38	21.84
	LTK hospital	13	11	84.62	1	9.09
	ZC hospital	30	25	83.33	6	24.00
Hospitals except the above five	Hospitals from other provinces	11	7	63.64	0	0
	Hospitals from other cities except Xingtai in Hebei	30	18	60.00	0	0
	Hospitals from counties except Shahe in Xingtai	142	91	64.08	0	0
	Hospitals except the above five in Shahe	236	153	64.83	0	0
Recipients with blood transfusion in more than 2 hospitals	Including the above five hospitals	10	9	90.00	2	22.22
	No including the above five hospitals	5	5	100.00	0	0
Unknown	Unknown	6	4	66.67	0	0
Total		799	592	74.10	92	15.54

**Table 3:** HIV infection among blood recipients between 1994 and 1998 in Shahe County.

Year	Blood recipients	HIV screening	Infected cases	Infection rate (%)
1994	128	95	7	7.36
1995	156	122	38	31.15
1996	134	95	14	14.74
1997	160	115	18	15.65
1998	189	136	11	8.09
Transfusion beyond the year^a^	32	29	4	13.79
Total	799	592	92	15.54

^a^Blood transfusion was carried out within at least 1 year between 1994 and 1998.

### Analysis of infection source

Among the 285 blood transfusion infection cases, 278 were not screened for HIV before transfusion and only 7 were screened as negative for HIV antibodies. Prior to 1998, different levels of medical institutions in Shahe County were required to obtain blood from the blood center of Xingtai City. However, most medical facilities did not implement this system, and the collection and supply of blood in some of these institutions was common owing to the poor management system. It was known that there was an overuse of blood transfusion at certain community medical institutions in Shahe. The main source of blood was from a mobile team of paid blood donors that could be booked by telephone if needed. There was no screening for HIV antibodies before blood donation. Blood donors on this team came from other regions such as Yunnan, Guangxi, Sichuan, Henan, Heilongjiang, Liaoning, and Jilin provinces. Donors had worked in local mines for many years and would donate blood when needed by a hospital. Among local residents of Shahe, a small number of blood donors also participated in paid blood donation.

During a census of local blood recipients in Shahe (November 2003 to February 2005), 37 local residents were found to have been blood donors between January 1, 1994 and December 31, 1998. Blood specimens were collected from these individuals and all 37 cases tested negative for HIV antibodies. Owing to a lack of blood donor registration information at local medical institutions, it was difficult to track and investigate the infection status of migrant workers in mining enterprises who came from other provinces, particularly those from the southern provinces of China where there was higher prevalence of HIV infection owing to injection drug use; Henan was another area affected by HIV infection among paid blood donors. In this case, we could not rule out the possibility that HIV-infected individuals participated in local paid blood donation in Shahe.

### Phylogenetic analysis of HIV-1 strains

**Fig 5:**
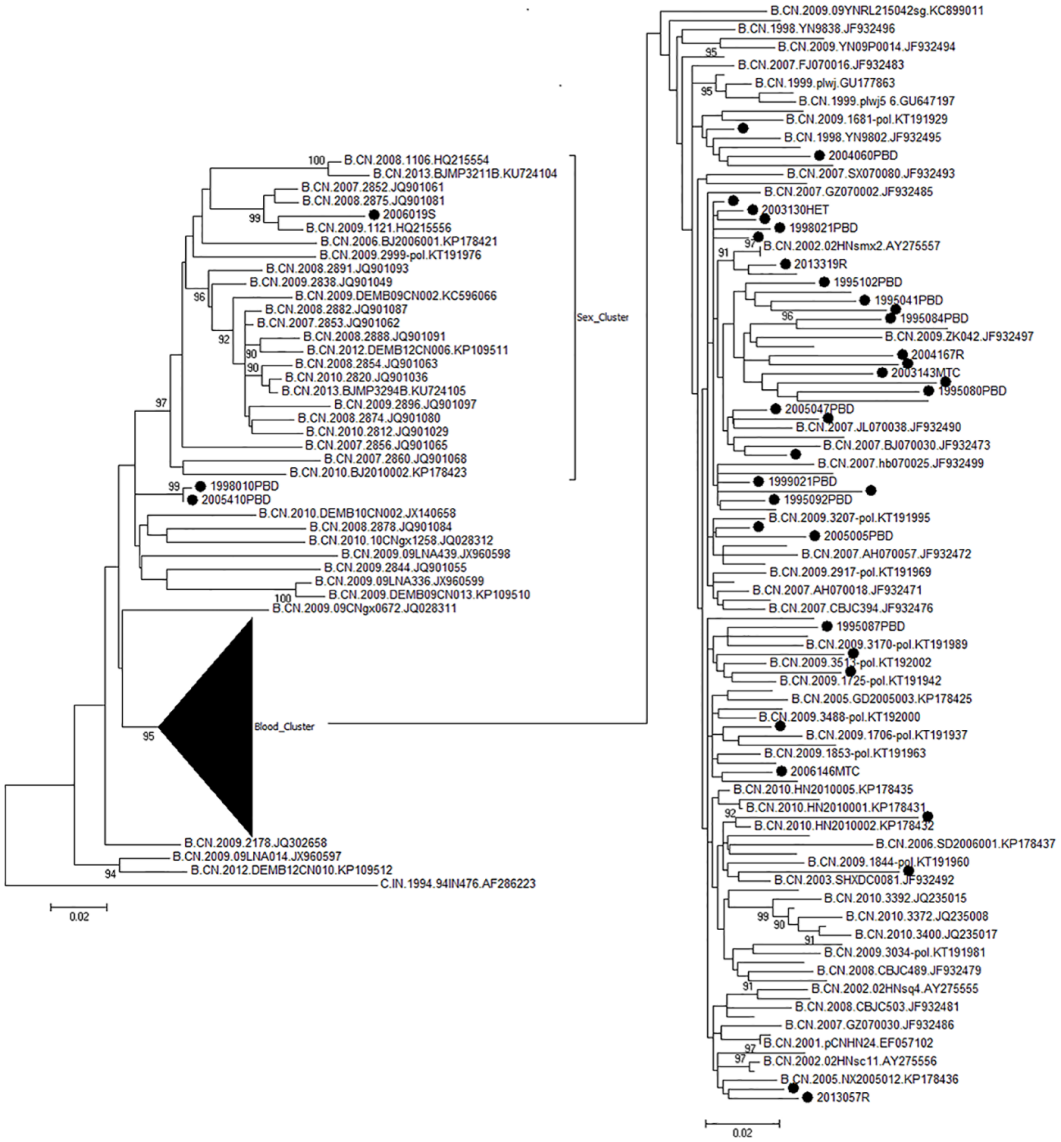
The connection between donors and recipients.

As shown in Fig 5, all prevalent HIV-1 strains among blood transfusion recipients were identified as HIV-1 subtype B′. In the ML tree, two clusters (Sex_Cluster and Blood_Cluster) were indicated. Within the Blood_Cluster, our sequences closely clustered together with the reference sequences from patients infected with HIV-1 via contaminated blood from Shandong, Shanxi, Henan, and so on; our sequences were far away from those patients (such as 2006019s) infected with HIV-1 through sexual contact within Sex_Cluster. The mean genetic distance within Blood_Cluster was 0.031±0.003, less than twice that (0.067±0.003) between the sexual transmission sequences, such as 2006019s, within Sex_Cluster and blood transmission sequences within Blood_Cluster. Moreover, the study sequences were also clustered with strains from Yunnan, such as from Ruili city and Dehong Prefecture. In particular, the root of the ML tree was the sequence from Dehong Prefecture in Yunnan. This indicated that blood transfusion cases were in fact related to the contaminated blood obtained from paid blood donors, and suggested that HIV-1 subtype B′ strain from Yunnan might have been introduced into Hebei through paid blood donors.

The international reference subtype B′ (●) was obtained from the HIV database (http://www.hiv.lanl.gov/content/index). Bootstrap values ≥90% are shown in the ML tree. The scale length indicates 2% nucleotide sequence divergence. Blood_Cluster and Sex_Cluster denote the blood transmission group and sexual transmission group, respectively. Within the sample ID and the reference sequence ID, PBD, R, MTC, HET, SD, SX, HN, NX, BJ, YN, and RL denote paid blood donor, recipient, mother to child, heterosexual, Shandong, Shanxi, Henan, Ningxia, Beijing, Yunnan, and Ruili of Yunnan. The donors and recipients were obtained from Langfang city and Xingtai city, respectively.

## Discussion

In 1989, an epidemic outbreak of HIV infection among injection drug users was first detected in Yunnan, China. Subsequently, the transmission of HIV/AIDS among this population was successively found in the Sichuan, Guangxi, and Xinjiang regions. Around 1995, migrant workers from Yunnan, Guangxi, Sichuan, Henan, and other provinces who worked in local mining enterprises of Shahe served as blood donors at county and township medical institutions. Poor local management of blood collection and supply, as well as no screening for HIV antibodies before blood donation, were the causes leading to an HIV/AIDS epidemic among local blood transfusion recipients in Shahe. In 1995, there was an outbreak of HIV infection among paid blood donors in the Gu’an and Yongqing regions of Langfang City. The area affected by HIV infection among blood donors in Langfang was about 400 km from Shahe. There were no indications that blood donors from Langfang had donated blood in Shahe city in Hebei. Nonetheless, given its close proximity, we could not rule out this possibility as a source of infection.

Molecular biological analysis showed that the prevalent HIV-1 strain was the B′ subtype in blood recipients from Shahe, similar to the B subtype found among injection drug users from Yunnan. The prevalent HIV-1 strain found among drug users from Yunnan belonged to the Thailand subtype B strain [6]. This suggests that the prevalent viral strains detected in blood recipients from Shahe originated from the Thailand subtype B in Yunnan. Our result is in agreement with previous findings by Yan et al. that the prevalent HIV-1 strain among blood recipients and donors from Guangdong Province was dominated by Thailand subtype B [7]; this is in line with the prevalent HIV-1 subtype among paid blood donors in Hebei Province[8].

In 1985, an epidemic of hepatitis C occurred among paid blood donors in China [9]; during 1993–1994, an epidemic of malaria emerged in this population [10]; and in 1995, an outbreak of HIV infection developed in the same group [2]. All these events were caused by cross-infection during plasmapheresis, a form of blood donation. HIV infection among paid blood donors was the direct cause of HIV infection among blood recipients. The blood HIV screening policy already specified that blood and blood products must be tested for HIV/AIDS in the “Provisions for the Monitoring and Control of HIV/AIDS”, approved by the State Council of China on December 26, 1987. In April 1988, the Ministry of Health promulgated the “Notice on Rectifying the Production Management of Blood Products”, which required the surveillance of blood product sources and testing of blood donors for HIV antibodies. On April 11, 1990, the Ministry of Health and the National Price Bureau issued the “Provisions for Strengthening the Work of Blood Transfusion”, which proposed unified blood source management as well as unified blood collection and supply, as well as only one blood station in each region (city), to improve the management of blood transfusion processes. In 1993, the Ministry of Health proposed the “Basic Standards for Blood Stations”, which required blood stations to perform second tests of all items, including testing of collected whole blood for HIV indicators. Since 1995, the Ministry of Health has required anti-HIV screening of all blood donors at blood stations. Our study results showed that the occurrence of blood transfusion recipients being infected with HIV-1 was associated with improper implementation of HIV screening policies and poor management of blood sources in local areas, particularly at community medical institutions. In 1998, the “Blood Donation Law” came into effect in China. A voluntary blood donation system was then put into practice and an HIV antibody screening system was widely implemented, resulting in a rapid decrease in transfusion-transmitted cases. In 2015, an HIV nucleic acid testing system was fully implemented and the risk of HIV infection through blood transfusion was thus further reduced.

Around 1995, government investment in medical institutions was inadequate and these facilities had to expand their medical income and reduce costs so as to maintain normal operation. Therefore, medical institutions commonly collected blood for use in patients admitted to their own facilities. Meanwhile, the scope of blood transfusion was also expanded. Particularly for pregnant women and during childbirth, community medical institutions generally used blood transfusion to treat postpartum hemorrhage, leading to a large number maternal HIV infections. Most of these medical institutions were located in rural areas and mainly served farmers, who were unaware that these facilities had violated the blood screening policies for transfusion recipients. Local governments ignored the situation and did not organize a census or blood screening until the appearance of HIV/AIDS in the region. From the perspective of the detection date of blood transfusion cases, there was a 9-year gap between the first detection date of HIV infection and the peak date of blood transfusion-related HIV/AIDS infections. This suggests that cases were detected relatively late, resulting in high rates of spousal transmission and MTCT. By contrast, HIV infections among paid blood donors were detected earlier in Hebei, which resulted in relatively low rates of spousal transmission and MTCT in that population [9]. At that point, hospital closings or a lack of adequate records management caused difficulties in tracking the sources of infection and investigation of the relevant evidence.

With regard to suspected blood transfusion infection date, only a few transfusion-related cases were detected from 1990 to 1994. Nevertheless, as inferred from the HIV infection dates of blood recipients in Shahe County, Xingtai City, HIV infections among paid blood donors in that city occurred earlier than among paid blood donors in Langfang [11], which further supports the possibility of transmission from migrant mining workers in Shahe who came from the southwest areas of China. According to the time distribution of the 285 blood transfusion-related HIV/AIDS cases in Hebei Province and time analysis of the cases at the outbreak site in Shahe, 1995 was the peak year of blood transfusion infections, and blood transfusion recipients had the highest HIV infection rates in that year. The second highest number of blood transfusion cases was recorded in 1996 and 1997, when HIV infection rates among blood transfusion recipients were second only to 1995.
